# Effect of whey protein on blood pressure in pre‐ and mildly hypertensive adults: A randomized controlled study

**DOI:** 10.1002/fsn3.1040

**Published:** 2019-04-21

**Authors:** Jing Yang, Hai‐Peng Wang, Xing Tong, Zeng‐Ning Li, Jia‐Ying Xu, Li Zhou, Bing‐Yuan Zhou, Li‐Qiang Qin

**Affiliations:** ^1^ Department of Clinical Nutrition The First Affiliated Hospital of Soochow University Suzhou China; ^2^ Department of Nutrition and Food Hygiene, School of Public Health Soochow University Suzhou China; ^3^ Department of Cardiovascular The First Affiliated Hospital of Soochow University Suzhou China; ^4^ Department of Clinical Nutrition The First Hospital of Hebei Medical University Shijiazhuang China; ^5^ State Key Laboratory of Radiation Medicine and Protection, School of Radiation Medicine and Protection Soochow University Suzhou China

**Keywords:** blood pressure, body composition, endothelial function, hypertension, obesity, whey protein

## Abstract

In China, the frequency of mild hypertension cases remains prevalently high. Meanwhile, diets containing functional ingredients that control blood pressure have received considerable attention. In this randomized, controlled intervention study, 65 participants were randomly assigned to consume 30 g of whey protein or maltodextrin daily for 12 weeks. Blood pressure, body composition, biochemical analysis in plasma, and flow‐mediated dilation (FMD), an index for evaluating endothelial function, were measured. Finally, 54 participants (27 participants in each group) completed the study. At the end of the intervention, the average systolic blood pressure (SBP) was 129.5 ± 7.7 mmHg in the control group and 128.2 ± 6.9 mmHg in the whey protein group (*p* = 0.052). In the overweight and obese participants, the SBP was significantly lower in the whey protein group than in the control group (126.5 ± 6.9 mmHg vs. 128.8 ± 7.4 mmHg, *p* = 0.033), and body fat, fat percentage, and waist circumference significantly decreased in the whey protein group (*p* = 0.010, 0.016, 0.019, respectively). No difference was observed between the control and whey protein groups with regard to the changes in plasma lipids, inflammatory cytokines, antioxidative indexes, endothelium‐1, nitric oxide, angiotensin II, and angiotensin‐converting enzyme. The increase in FMD was significantly higher in the whey protein group than in the control group (5.2% vs. 0.3%, *p* = 0.040). In conclusion, whey protein significantly decreased SBP in pre‐ and mildly hypertensive adults, who are also overweight and obese. Whey protein also improved endothelial function. The lowering effect of blood pressure was probably related to body fat loss in these participants.

## 1 INTRODUCTION

Cardiovascular diseases remain the major cause of death worldwide, and hypertension has been identified as a large but modifiable risk factor for cardiovascular mortality (Brunström & Carlberg, 2018). In 2015, the global age‐standardized prevalence of hypertension is 24.1% in men and 20.1% in women (NCD Risk Factor Collaboration, 2017). Although blood pressure decreased substantially in high‐income countries, it might have increased in some areas such as east and southeast Asia. In China, the age‐standardized and sex‐standardized prevalence of hypertension is 37.2%, and 66% of the cases is mild hypertension (Lu et al., 2017). Obesity is accompanied by several hormonal, inflammatory, and endothelial alterations. These alterations induce a stimulation of several mechanisms that contribute to hypertension (Seravalle & Grassi, 2017).

A large proportion of patients with mild hypertension do not have the ideal blood pressure control because of their poor medication compliance; thus, a supplementary nonpharmacological intervention is necessary (Lu et al., 2017). Diets with ingredients that aid in maintaining a normal blood pressure have received a considerable amount of attention from researchers and the public. Whey protein was found to lower blood pressure in animal studies and human trials, with inconsistent results (Pal & Radavelli‐Bagatini, 2013). However, all human trials using whey protein concentrate/isolate as intervention were conducted in the Western population, who were under resistance exercise, hypocaloric diet, or various health conditions (Claessens, van Baak, Monsheimer, & Saris, 2009; Fekete, Giromini, Chatzidiakou, Givens, & Lovegrove, 2016; Flaim, Kob, Di Pierro, Herrmann, & Lucchin, 2017; Hodgson et al., 2012; Kjølbæk et al., 2017; Pal & Ellis, 2010). The main purpose of the present study was to examine the effect of intact whey protein on the blood pressure of pre‐ and mildly hypertensive Chinese adults without restriction on energy diet or exercise. Apart from the blood pressure, the secondary outcomes including body composition, plasma biochemical markers, and vascular endothelial function were measured.

## 2 METHODS

The trial was a randomized controlled intervention study and was conducted at the First Affiliated Hospital of Soochow University between September 2015 and October 2015. Pre‐ and mildly hypertensive adults who were not taking antihypertensive or cholesterol‐lowering medications were recruited from the community of Suzhou, Jiangsu, China. The participants were more than 18 years of age. According to 2010 Chinese guidelines for the management of hypertension (Writing Group of, 2010 Chinese Guidelines for the Management of Hypertension 2011), the participants had systolic blood pressure (SBP): 130–159 mmHg or diastolic blood pressure (DBP): 80–99 mmHg. Participants were excluded on the basis of the following criteria: has lactose intolerance; alcoholism; has mental abnormalities; are pregnant or lactating; has a history of chronic diseases including coronary heart disease, diabetes, liver and/or kidney diseases, tumor, dyslipidemia, sarcoidosis, hyperthyroidism, and cerebrovascular disease. Participants using supplements, such as protein powder, fish oil, antioxidants, and anti‐inflammatory products, were also excluded. The trial was approved by the Ethics Committee of the First Affiliated Hospital of Soochow University and was registered at the Chinese Clinical Trial Registry with the registration number of ChiCTR‐IPR‐15005756. A written inform of consent was obtained from each participant.

The recruited participants were randomly divided into the whey protein group and the control group with a random sequence software. Whey protein concentrate 80 was purchased from Milk Specialties Global. Maltodextrin (Shijiazhuang Huachen Starch Sugar Production Co., Ltd) was used as the control. Whey protein and maltodextrin were packed into identical foil sachets, and each sachet contained 15 g of powder. The participants were instructed to consume one sachet of powder mixed with approximately 200 ml of water twice per day with meal. To monitor intervention compliance, the participants were asked to record the number of the sachets and to keep the empty packages. All participants were asked to maintain their lifestyle and eating habits. At 12 weeks, the dietary intake was assessed using a 72‐hr food recall. The food intake data were analyzed based on the Chinese Food Composition Database through a computer software.

A follow‐up was done to each participants every week. Blood pressure was measured at the start (0 or baseline), 4th, 8th, and 12th week of the trial. The participants were asked to sit quietly for at least 15 min, and the blood pressure was measured three times with 5‐min intervals using an automatic blood pressure measuring device (Pro Doctor BP3AJ1‐1R; Microlife). When the blood pressure values varied by ≥10 mmHg, additional measurements were taken.

At the baseline and the end of the trial, body composition measurement, biochemical analysis in plasma, and flow‐mediated dilation (FMD), an index for evaluating endothelial function (Korkmaz & Onalan, 2008), were performed. In brief, the body weight was taken by having the participants wear light clothes and no shoes; height was measured to the nearest 0.5 cm with a wall‐mounted stadiometer. Waist circumference was determined by placing a measuring tape around the bare stomach at midpoint between the costal margin and the anterior superior iliac spine. Body mass index (BMI) was calculated as weight (kg)/[height (m)]^2^. The BMI classification is as follows: ≤18.5 kg/m^2^ underweight; 18.5–23.9 kg/m^2^ normal weight; 24–27.9 kg/m^2^ overweight; and ≥28 kg/m^2^ obese. Body fat mass, body fat percentage, body muscle mass, and muscle tissue were evaluated through bioelectrical impedance analysis (The InBody S10 Body Composition Analyzer).

For the biochemical analysis, venous blood samples were collected after an overnight fasting of at least 10 hr. The plasma was separated through centrifugation and then stored at −80°C. The plasma levels of glucose, lipids (cholesterol, triglyceride, high high‐density lipoprotein cholesterol, and low high‐density lipoprotein cholesterol), and high‐sensitivity C‐reactive protein (hsCRP) were measured with an automatic chemistry analyzer (AU5400; Olympus Corporation). The kits for glucose and lipids were purchased from Sekisui Medical Technology (China) LTD. The kit for CRP was purchased from Orion Diagnostica Oy. The plasma levels of angiotensin‐converting enzyme (ACE), angiotensin II, nitric oxide (NO), and vascular endothelium‐1 (ET‐1) were analyzed using commercial test kits (Abcam). Inflammation markers of tumor necrosis factor‐⍺ (TNF‐⍺) and interleukin‐6 were evaluated via flow cytometry (FACSCalibur). Antioxidant marker superoxide dismutase (SOD) and peroxide marker malondialdehyde (MDA) were analyzed by spectrophotometry (Shanghai Lengguang Technology Co. Ltd).

Flow‐mediated dilation was performed by a single trained researcher using an iE33 Ultrasound System (Philips). The participants were instructed to lie down in the supine position and open their right arm 15° outwards. The inner diameter of the brachial artery was measured 2 cm above the elbow socket at the most obvious artery pulse at the end of diastole (D0). The blood pressure was maintained at 30 mmHg above the SBP for 5 min. The diameter of the largest brachial artery was measured again at the same place (D1). The change of the brachial artery diameter after reactive hyperemia was the endothelial‐dependent FMD (%) = (D1 − D0)/D0 × 100%.

All statistical analyses were performed with the SPSS statistical software (version 19.0; IBM Corp). Changes in body composition, biochemical indexes, and FMD from baseline to the end of trial were calculated. The differences between groups were analyzed by using the independent sample *t* test. Suitable checks for normality were performed, and data were logarithmically transformed as appropriate. *p* Values below 0.05 were considered significant. Data presented in the text, tables, and figures represent means ± standard deviations (SDs).

## 3 RESULTS

**Figure 1:**
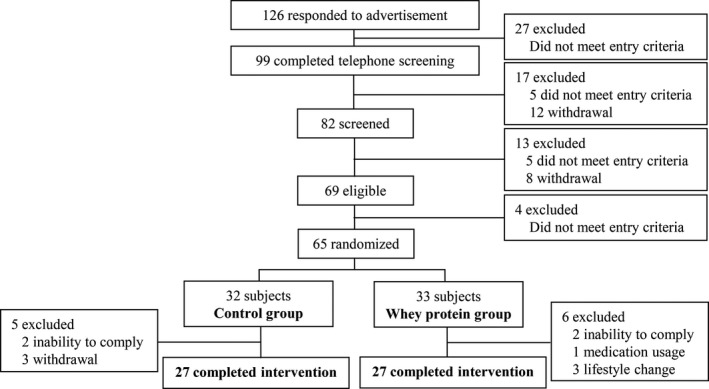
Flowchart of subject inclusion

A total of 65 participants with pre‐ and mild hypertension were included in the trial and were randomly assigned to the control group (32 participants) or whey protein group (33 participants). In the control group, five participants dropped out because of poor intervention compliance or personal reasons not related to the study. In the whey protein group, six participants were excluded because of poor intervention compliance, their use of blood pressure‐lowering medication, or obvious changes in their diet and exercise. Finally, 54 (27 participants in each group) completed the study (Figure 1).

Among the 54 completing participants, 33 were male. The age ranged from 22 to 69 years with mean of 42.6 years. The range of SBP was 117–148 mmHg (mean: 132.8 mmHg) and DBP was 70–99 mmHg (mean: 87.0 mmHg). SBP and/or DBP of participants fall in the range of pre‐ and mild hypertension. The subgroups were well balanced for baseline characteristics such as gender, age, BMI, and blood pressure (Table 1).

**Table 1:** Baseline characteristics of study subjects (mean ± *SD*)

	Control	Whey protein	*p*
Male/female (*n*)	16/11	17/10	0.83
Age (year)	43.84 ± 11.76	42.37 ± 11.60	0.66
Weight (kg)	68.67 ± 12.19	68.26 ± 10.99	0.89
BMI (kg/m^2^)	24.33 ± 2.39	24.19 ± 3.10	0.85
Body fat (kg)	16.38 ± 4.50	17.15 ± 6.05	0.60
Body fat percentage (%)	24.20 ± 6.55	24.84 ± 7.20	0.73
Lean tissue (kg)	52.30 ± 11.34	51.11 ± 8.30	0.66
SBP (mmHg)	132.27 ± 8.32	133.30 ± 6.49	0.62
DBP (mmHg)	86.73 ± 5.96	87.35 ± 5.02	0.68

Abbreviations: BMI, body mass index; DBP, diastolic blood pressure; SBP, systolic blood pressure.

We further divided the participants according to BMI. Twenty‐four participants (12 in control group and 12 in whey protein group) were normal in weight, whereas the other 30 participants (15 in control group and 15 in whey protein group) were overweight and obese. In the normal weight participants, the changes in body composition were not significantly different between the control and whey protein groups. In the overweight and obese group, the body fat, fat percentage, and waist circumference significantly decreased in the whey protein group compared with the control group (*p* = 0.010, 0.016, 0.019, respectively). The whey protein intervention further decreased the body fat by 1.25 kg and the waist circumference by 1.69 cm when considering the change in the control group. Despite the loss of weight and BMI in the whey protein group, the values were not statistically different from those of the control group. Lean tissue and muscle tissue decreased in the control group and increased in the whey protein group, but no significant difference was observed (Table 2).

**Table 2:** Change in body compositions before and after the intervention stratified by BMI status (mean ± *SD*)

	Normal weight		Overweight and obesity	
	Control (*n* = 12)	Whey protein (*n* = 12)	Control (*n* = 15)	Whey protein (*n* = 15)
Weight (kg)	0.00 ± 0.93	−0.40 ± 0.67	−0.49 ± 1.37	−1.33 ± 0.82
BMI (kg/m^2^)	0.00 ± 0.38	−0.12 ± 0.25	−0.17 ± 0.46	−0.47 ± 0.29
Body fat (kg)	0.06 ± 1.08	−0.57 ± 0.75	−0.19 ± 0.91	−1.44 ± 0.89*
Body fat percentage (%)	0.18 ± 1.54	−0.83 ± 1.3	−0.19 ± 0.93	−1.46 ± 0.98*
Waist circumference (cm)	0.04 ± 1.21	−0.28 ± 1.54	−0.04 ± 1.09	−1.73 ± 1.51*
Lean tissue (kg)	−0.06 ± 0.68	0.17 ± 0.92	−0.30 ± 0.69	0.11 ± 0.68
Muscle tissue (kg)	−0.06 ± 0.64	0.16 ± 0.87	−0.28 ± 0.65	0.11 ± 0.64

Abbreviation: BMI, body mass index.

* *p* < 0.05 compared with control group.

The daily intake of protein from diets (not included from the intervention) was 65.5 ± 11.4 g in the control group and 64.8 ± 13.4 g in the whey protein group (*p* = 0.85). Other nutrients and energy intake were also similar between the two groups (Table 3). No significant difference in nutrient and energy intake was found when the participants were divided according to BMI.

**Table 3:** Energy and nutrient intake assessed at week 12 between the whey protein and control groups (mean ± *SD*)

	Control (*n* = 27)	Whey protein (*n* = 27)	*p*
Energy (kcal)	1908.84 ± 303.13	1910.24 ± 298.05	0.98
Protein (g)	65.47 ± 11.44	64.81 ± 13.42	0.84
Total fat (g)	61.69 ± 19.87	60.97 ± 21.93	0.90
Carbohydrate (g)	245.29 ± 47.34	246.34 ± 48.17	0.92
Calcium (mg)	475.38 ± 197.80	484.94 ± 234.61	0.87
Sodium(mg)	469.77 ± 174.75	458.36 ± 214.97	0.83
Potassium (mg)	1586.84 ± 435.10	1661.90 ± 444.17	0.53

**Figure 2:**
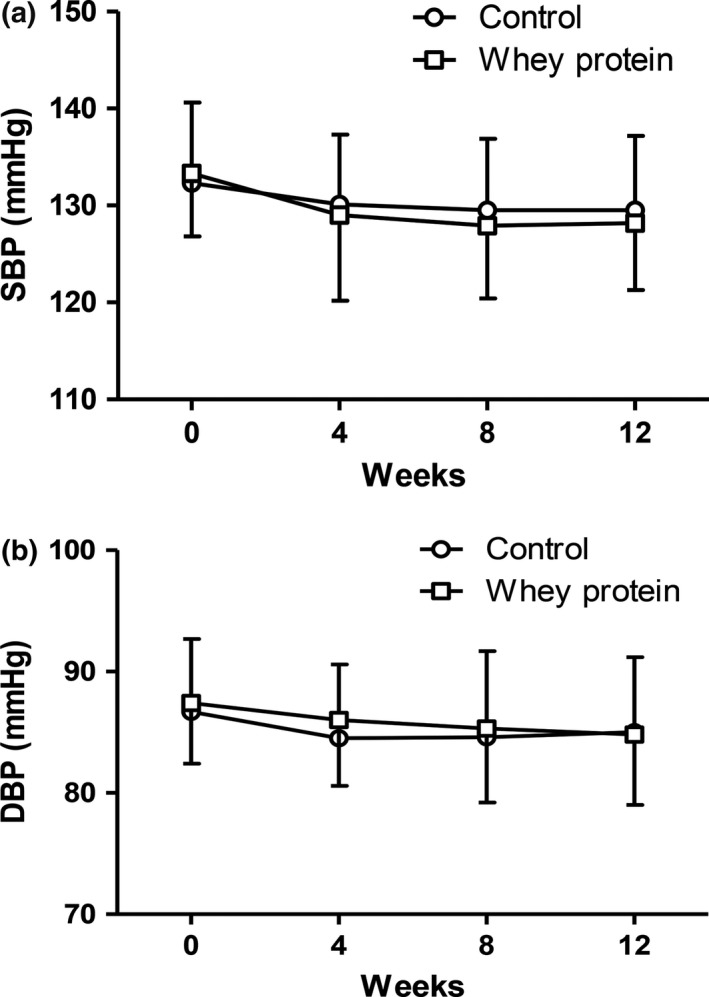
Changes in blood pressure between the whey protein group and control group during the intervention. (a) Systolic blood pressure; (b) diastolic blood pressure

**Figure 3:**
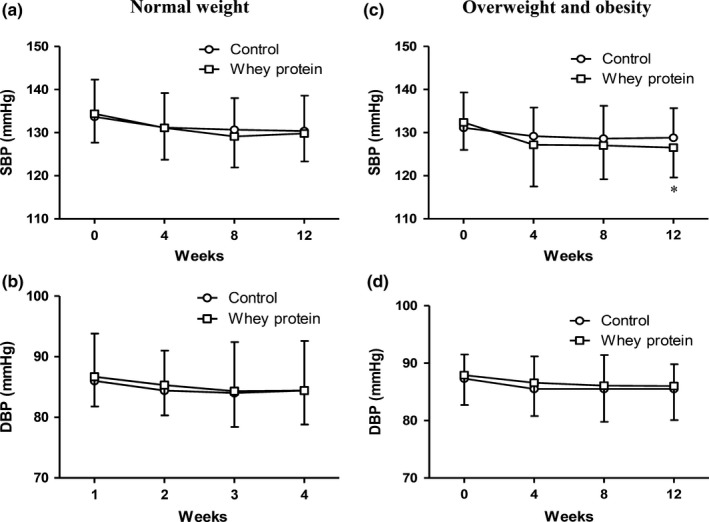
Changes in blood pressure over time between the whey protein and control groups stratified by BMI status. (a, b) Normal weight; (c, d) overweight and obesity. *p < 0.05 compared with control group

A tendency for lower SBP and DBP existed during the intervention. At the end of the trial, SBP was 129.5 ± 7.7 mmHg in the control group and 128.2 ± 6.9 mmHg in the whey protein group, with a borderline statistical significance (*p* = 0.052). DBP was similar between the control group (85.0 ± 6.2 mmHg) and the whey protein group (84.8 ± 5.8 mmHg) (Figure 2). After dividing according to BMI, still no difference in blood pressure was found in the normal weight participants. However, SBP was significantly lower in the whey protein group than in the control group (126.5 ± 6.9 mmHg vs. 128.8 ± 7.4 mmHg, *p* = 0.033) in the overweight and obese participants. No significant change in DBP was observed (Figure 3).

No difference between the control and whey protein groups was observed with regard to the changes in the levels of biochemical markers including lipids, glucose, inflammatory cytokines, antioxidative indexes, ET‐1, NO, angiotensin II, and ACE. The similar results were observed when the participants were divided according to BMI (Table 4). In the overweight and obese participants, whey protein obviously increased NO level without a significant difference between the two groups owing to high interindividual variation.

**Table 4:** Change in biochemical markers before and after the intervention stratified by BMI status (mean ± *SD*)

	Normal weight		Overweight and obesity	
	Control (*n* = 12)	Whey protein (*n* = 12)	Control (*n* = 15)	Whey protein (*n* = 15)
Total cholesterol (mmol/L)	−0.14 ± 0.71	−0.15 ± 0.72	−0.20 ± 0.43	−0.36 ± 0.58
HDL‐C (mmol/L)	−0.13 ± 0.13	−0.15 ± 0.26	0.03 ± 0.10	0.00 ± 0.26
LDL‐C (mmol/L)	−0.15 ± 0.43	−0.10 ± 0.47	−0.18 ± 0.32	−0.32 ± 0.40
Triglycerides (mmol/L)	0.38 ± 0.49	0.26 ± 0.44	0.01 ± 0.25	0.20 ± 0.97
Glucose (mmol/L)	−0.44 ± 0.43	−0.38 ± 0.51	−0.61 ± 0.40	−0.54 ± 0.43
hsCRP (mg/L)	0.02 ± 0.35	0.49 ± 2.46	−0.29 ± 3.68	−0.27 ± 3.05
TNF‐α (pg/ml)	0.11 ± 1.99	0.36 ± 1.26	0.15 ± 1.34	0.02 ± 0.83
IL‐6 (pg/ml)	0.20 ± 1.99	−0.06 ± 2.80	0.07 ± 2.01	−0.04 ± 1.95
SOD (U/ml)	5.62 ± 5.50	3.05 ± 6.51	−2.15 ± 5.81	−3.81 ± 5.02
MDA (nmol/ml)	−0.56 ± 1.88	−2.61 ± 4.95	−2.52 ± 3.86	−1.44 ± 2.76
ACE (ng/ml)	−0.84 ± 9.76	−0.69 ± 9.42	3.53 ± 11.87	2.03 ± 7.63
AngII (ng/ml)	−0.12 ± 0.40	−0.43 ± 1.87	−0.11 ± 0.49	−0.08 ± 0.43
ET‐1 (pg/ml)	0.37 ± 5.70	−0.08 ± 5.48	−4.92 ± 12.96	−4.67 ± 8.38
NO (µmol/L)	−0.39 ± 1.66	−0.74 ± 1.28	−0.13 ± 1.92	1.55 ± 2.68

Abbreviations: ACE, angiotensin‐converting enzyme; AngII, angiotensin II; ET‐1, vascular endothelium‐1; HDL‐C, high high‐density lipoprotein cholesterol; hsCRP, high‐sensitivity C‐reactive protein; LDL‐C, low high‐density lipoprotein cholesterol; IL‐6, interleukin‐6; MDA, malondialdehyde; NO, nitric oxide; SOD, superoxide dismutase; TNF‐α, tumor necrosis factor‐α

**Figure 4:**
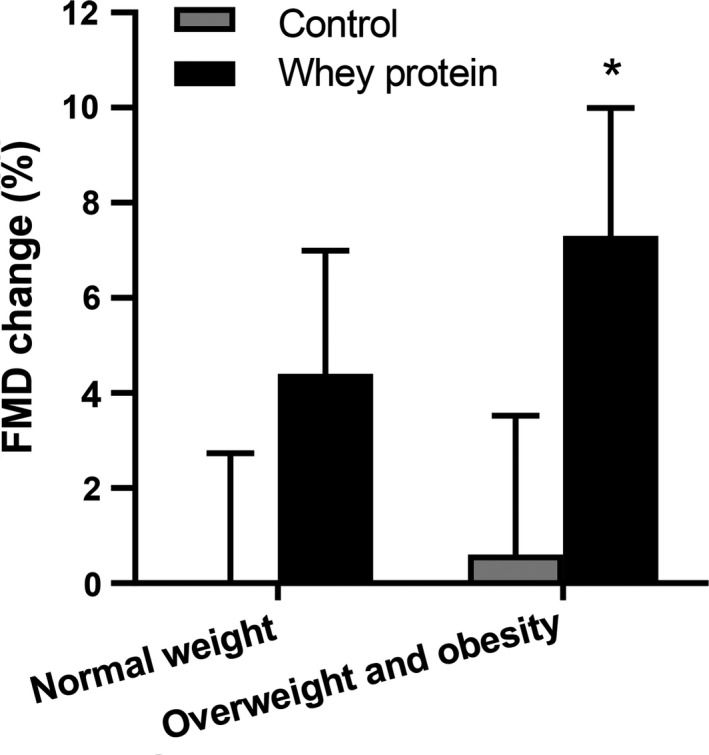
Changes in flow‐mediated dilation (%) before and after the intervention between the whey protein and control groups. *p < 0.05 compared with control group

The FMD index was normally distributed. The FMD increased by 5.2% in the whey protein group and 0.3% in the control group (*p* = 0.040). In the normal weight participants, no significant difference in FMD change was found between the two groups. In the overweight and obese participants, the FMD had a significantly higher increase in the whey protein group than in the control group (7.3% vs. 0.6%, *p* = 0.046) (Figure 4).

## 4 DISCUSSION

The effect of whey protein or peptide on blood pressure of participants with abnormal blood pressure was investigated in three trials. In the Fekete study, intact whey protein supplementation for 8 weeks significantly decreased 24‐hr ambulatory SBP and DBP in adults with pre‐ and mild hypertension (Fekete et al., 2016). Fluegel also found that whey beverage consumption for 6 weeks significantly decreased SBP and DBP in adults with elevated blood pressure (Fluegel et al., 2010). However, Lee indicated that the regular consumption of a milk drink containing whey peptides for 12 weeks has no effect on blood pressure in mildly hypertensive individuals (Lee, Skurk, Hennig, & Hauner, 2007). Our study found that the effect of whey protein on blood pressure was dependent on body weight because a significant decrease in SBP was observed only in the overweight and obese participants. Compared with our study, the participants in the Pal study had higher average BMI (31.3 kg/m^2^) and slightly lower blood pressure (SBP 119.3 mmHg). They found that the SBP significantly decreased by 4% after a 12‐week consumption of whey protein; however, no significant difference was observed between whey the protein and control groups (Pal & Ellis, 2010). In the Flaim study with obese (average BMI: 37.1 kg/m^2^) diabetic patients, no change in blood pressure was observed after a 12‐week intervention with whey protein (Flaim et al., 2017), even though they were the similar baseline blood pressure (average SBP: 133.3 mmHg) with our study.

To investigate the effect of body weight on blood pressure, we divided the participants according to BMI and further compared the change in body composition between the control and whey protein groups. In fact, whey protein is the primary ingredient in most protein powders and is primarily used by athletes and bodybuilders to increase muscle strength and decrease body fat mass. In a meta‐analysis including nine randomized controlled trials, whey protein significantly decreased body weight and fat mass in overweight and obese participants (Wirunsawanya, Upala, Jaruvongvanich, & Sanguankeo, 2018). In the present study, whey protein beneficially affected body fat more than lean tissue in the overweight and obese participants. This result is consistent with the Flaim study, where whey protein intervention significantly improved fat mass (Flaim et al., 2017). In our recent animal study, chronic whey protein supplementation significantly decreased lipid droplets in the liver without affecting the body weight in ApoE^−/−^ mice (Zhang et al., 2018). Enhancing satiety and suppressing appetite is the major mechanism of improving body composition. Hall found that higher postprandial circulating levels of amino acids were associated with increased satiety and suggested that fast proteins, such as whey protein, were more satiating than slow proteins (Hall, Millward, Long, & Morgan, 2003). Pesta also found that whey protein consumption obviously increased the levels of satiety hormones, including glucagon‐like peptide‐1, dipeptidyl peptidase 4, and cholecystokinin. Protein has also been found to have the highest diet‐induced thermogenesis (DIT) values among carbohydrates and fats in diet (Pesta & Samuel, 2014). The increase in DIT may increase satiety by the expansion of oxygen demand for protein metabolism, which can potentially enhance satiety (Pesta & Samuel., 2014; Westerterp‐Plantenga, Rolland, Wilson, & Westerterp, 1999).

Whey protein contains ACE‐inhibitory peptide, blocking the enzymatic conversion of angiotensin I to vasoconstrictor angiotensin II, and lowering blood pressure. ACE inhibition has become an established principle in the treatment of hypertension in clinical medicine (Messerli, Bangalore, Bavishi, & Rimoldi, 2018). We assume that whey protein can be degraded into active peptides in human gut and exert antihypertensive effects. However, we did not observe obvious change in the serum levels of ACE and angiotensin II. The possible explanations included the degradation of bioactive peptides by intestinal or plasma peptidases, or insufficiency in intestine absorbed. In fact, in vitro ACE‐inhibiting activity does not always correlate with antihypertensive activity. In Marques' study, despite presenting ACE‐inhibitory activity, whey protein hydrolysate and fractions did not significantly reduce the blood pressure of SHR after single oral administration (Marques et al., 2015).

The improvement of lipid profile, inflammatory cytokines, and antioxidative indexes is believed to contribute to blood pressure control. Inflammation markers are also associated with endothelial function (Ballard et al., 2013; Da Silva, Bigo, Barbier, & Rudkowska, 2017). In the present study, no difference was observed between the lipids, glucose, inflammatory cytokines, and antioxidative indexes of the two groups. In agreement with our study, Flaim found that whey protein intervention did not affect oxidative damage, inflammation, and glucose levels although fat mass was significantly decreased (Flaim et al., 2017). However, a trial with pre‐ and mildly hypertensive adults showed that whey protein significantly decreased serum total cholesterol and borderline decreased triacylglycerol (Fekete et al., 2016). Overall, animal studies and human trials relating to whey protein intervention in the above indexes yielded inconsistent results owing to variation in study designs and observational subjects (Avila et al., 2018; Ballard et al., 2013; Claessens et al., 2009; Da Silva et al., 2017; Fekete et al., 2016; Flaim et al., 2017; Hodgson et al., 2012; Kjølbæk et al., 2017; Pal & Ellis, 2010; Tranberg, Madsen, Hansen, & Hellgren, 2015; Zhang et al., 2018).

A delicate balance between vasoconstriction and vasodilation plays a pivotal role in endothelial function and then blood pressure regulation. Vasoconstrictor ET‐1 and vasodilator NO participate in the modulation of the endothelial function. After a 24‐hr incubation period, whey protein hydrolysate attenuated the TNF‐α‐induced endothelial inflammation by normalizing TNF and gene expression of endothelial NO synthase in human umbilical vein endothelial cells (Da Silva et al., 2017). We did not observe the changes in plasma NO and ET‐1, which is assistant with our previous animal study (Zhou et al., 2017). NO and ET‐1 mainly exert paracrine and autocrine effects (Ballard et al., 2013). Thus, the serum levels of NO and ET‐1 may not completely reflect its bioactivity.

In the present study, we found that whey protein intervention significantly improved FMD, which is the gold standard for evaluating endothelial function (Korkmaz & Onalan, 2008). The beneficial effect was evidenced in the participants with impaired brachial artery FMD where ingestion of whey‐derived extract significantly increased postprandial FMD measured at 30 and 120 min postingestion (Ballard et al., 2013). In the Fekete's study, whey protein improved FMD by ameliorating the 24‐hr ambulatory blood pressure with no effect on any inflammatory markers or on plasma nitric oxide and serum ACE activity (Fekete et al., 2016). However, the association between FMD and blood pressure needs further validation (Liu et al., 2017).

Our study has several limitations. First, only 54 participants were included in the trial. It cannot delete the possibility that the sample size was small to reach statistical significance. Second, the amount of whey protein in our study was 30 g/day, which was less than the amount (>50 g/day) used in studies from Western countries (Claessens et al., 2009; Fekete et al., 2016; Pal & Ellis, 2010). The daily protein intake recommended by the Chinese Nutrition Association is 55 g for women and 65 g for men in light physical activity (Chinese Nutrition Society, 2016). The protein intake from diets generally needed this recommendation. Although a higher amount might increase the chance of observing the positive change in blood pressure, we selected a more practical intervention amount in the present study. Third, maltodextrin, as a control, might have additional effects on the outcomes we observed. Lastly, white coat hypertension phenomenon might influence the blood pressure measurement.

In conclusion, whey protein significantly decreased SBP in pre‐ and mildly hypertensive adults with overweight and obesity. Whey protein also improved endothelial function. The effect of whey protein on body weight and body composition, rather than on lipid profile, inflammatory responses, and anti‐oxidation, plays an important role in the modulation of blood pressure.

## CONFLICT OF INTEREST

The authors declare no conflict of interest.

## ETHICS STATEMENT

This study conforms to the Declaration of Helsinki. All protocols and procedures were ethically reviewed and approved by the Ethic Committee of the First Affiliated Hospital of Soochow University. Written and verbal informed consent was collected from all participants prior to participation.
